# Alaska Dental Health Aide Program

**DOI:** 10.3402/ijch.v72i0.21198

**Published:** 2013-08-05

**Authors:** Sarah Shoffstall-Cone, Mary Williard

**Affiliations:** 1 DENTEX Clinical Site Director with the Alaska Native Tribal Health Consortium; 2 Director, Department of Oral Health Promotion and Director, Dental Health Aide Therapist Educational Program with the Alaska Native Tribal Health Consortium

**Keywords:** dental workforce, dental mid-level providers, dental therapist

## Abstract

**Background:**

In 1999, An Oral Health Survey of American Indian and Alaska Native (AI/AN) Dental Patients found that 79% of 2- to 5-year-olds had a history of tooth decay. The Alaska Native Tribal Health Consortium in collaboration with Alaska's Tribal Health Organizations (THO) developed a new and diverse dental workforce model to address AI/AN oral health disparities.

**Objectives:**

This paper describes the workforce model and some experience to date of the Dental Health Aide (DHA) Initiative that was introduced under the federally sanctioned Community Health Aide Program in Alaska. These new dental team members work with THO dentists and hygienists to provide education, prevention and basic restorative services in a culturally appropriate manner.

**Results:**

The DHA Initiative introduced 4 new dental provider types to Alaska: the Primary Dental Health Aide, the Expanded Function Dental Health Aide, the Dental Health Aide Hygienist and the Dental Health Aide Therapist. The scope of practice between the 4 different DHA providers varies vastly along with the required training and education requirements. DHAs are certified, not licensed, providers. Recertification occurs every 2 years and requires the completion of 24 hours of continuing education and continual competency evaluation.

**Conclusions:**

Dental Health Aides provide evidence-based prevention programs and dental care that improve access to oral health care and help address well-documented oral health disparities.

The Alaska Native Tribal Health Consortium (ANTHC), founded in 1997, is a non-profit, statewide organization that provides a range of medical and community health services for more than 125,000 Alaska Natives. It is part of the Alaska Tribal Health System, which is owned and managed by the 229 federally recognized tribes in Alaska and by their respective regional health organizations. In alignment with the organizational mission of “Providing the highest quality health services in partnership with our people and the Alaska Tribal Health System”, and working toward our corporate vision of “Alaska Natives are the healthiest people in the world”, it was a natural outgrowth of this work for ANTHC to include as a part of their strategic plan the development of the Dental Health Aide (DHA) Program to supply dental providers for our rural villages and regional centers which are grossly underserved. The DHA program addresses the need to improve access to a health profession workforce that is skilled, diverse, and culturally competent.

Alaska's American Indian/Alaska Native (AI/AN) population experiences on-going oral health disparities. This is evident from data drawn from both National and State assessments. In an Indian Health Service survey conducted in 1999 children aged 2–5 years have almost 5 times the amount of tooth decay as do children in the same age range in the rest of the United States. Native children aged 6–14 years have 4.5 times the amount of untreated decay in their permanent teeth than children in the rest of the United States. 92% of teenagers (aged 15–19) have a history of early periodontal disease and 57% have untreated tooth decay. Older adults have major problems also with 51% having untreated decay and 26% having lost all of their teeth (ages 55 +) (1). According to the article *The Oral Health Status of American Indian/Alaska Native Preschool Children: a Crisis in Indian Country*, “the percentage of children with untreated decay was more than 3 times higher in AI/AN children compared to the NHANES III children (68 vs. 19%)” (2). Similar data exists for older children and adults. Findings from the 2010/2011 State of Alaska Dental Assessment showed 83.4% of AI/AN third graders had experienced caries, 39.5% of these children had untreated decay (3). These percentages indicate that schools and communities are populated with children who have untreated oral infections. Ultimately, this situation leads to a demand for costly urgent care: in 2004–2006, the Alaska Native Tribal Health Consortium (ANTHC) Department of Environmental Health and Engineering research found that in one village alone, 17 of 21 (81%) 4- to 6-year-olds had received oral surgery under general anaesthesia to treat severe early childhood caries (ECC). The average cost per operating room surgical procedure was $7,433 (4). Alaska's overwhelming oral health disparities will continue to increase without a comprehensive, long-term delivery structure to implement evidence-based prevention strategies. To begin a successful campaign to reduce oral health disparities, a multi-tiered, prevention-based approach to care has been designed. The focus is creating a culture of participation in oral health preventive practices, and to augment support for existing dental teams.

Although Alaska has the largest landmass in the US, there are just 710,231 residents. 14.8% of Alaskans are AI/AN (5). There are 215 villages spread throughout Alaska, with the vast majority accessible only by boat, bush plane or snowmobile. Many of these communities receive no on-site dental services. Regional Tribal Health Organization dental departments have historically provided care through itinerant visits to villages in their area from the regional hub. The frequency of dental visits depends on factors such as geography, weather and the availability of a dentist. The priority for services during these itinerant visits is children.

By early 2000, sobering statistics about Alaska Natives’ oral health and the chronic difficulties in staffing professional dental services led executives from across the Alaska Tribal Health System to concentrate on opportunities to develop a better system of oral health care delivery in Alaska. From these discussions arose the Alaska Dental Health Aide Initiative, a multifaceted approach to increase both the number of dental providers in rural Alaska and the level of dental services available to Alaska Native people. The resulting Dental Health Aide (DHA) Program is modeled after and part of the Community Health Aide/Practitioner (CHAP) Program, created in the late 1960s in response to the poor health status of rural Alaska Natives. The CHAP program has operated successfully in Alaska for 50 years.

The DHA program, like the CHAP program, selects AI/AN people with strong ties to their communities and provides them with basic health care education. The DHA program includes 4 types of dental care providers. The Primary Dental Health Aide (PDHA) concentrates on delivering preventive services at the village level. The Expanded Function Dental Health Aide (EFDHA) has an elevated skill set that enables their function under the direct or indirect supervision of a dentist and their performance of simple to complex tooth restorations and supra-gingival dental cleanings. The Dental Health Aide Hygienist (DHAH) is able to administer local anaesthetic. The highest level of provider is the Dental Health Aide Therapist (DHAT), a dental provider, similar to a Physician Assistant in the field of medicine. While the DHAT is a new type of provider in the United States, DHAT-like providers work in over 50 countries worldwide, including Canada and New Zealand. These new Alaska dental team members work with the THO dentists and hygienists to provide prevention, basic restorative, and urgent care services. The Alaska Tribal Health System currently has 58 DHAs. There are 25 certified Dental Health Aide Therapists, 1 Dental Health Aide Hygienist, 8 Expanded Function Dental Health Aides and 24 Primary Dental Health Aides working in tribal programs around the state. These new providers working in tandem with their supervising dentists provide a framework to institute effective dental disease prevention programs which were never before possible in Alaska's rural communities. Best practice in dental prevention include gathering data to make accurate needs assessments, multiple interventions during the year for high risk patients, and motivational interviewing techniques to support and sustain behavior changes toward healthier habits. These best practices are part of the philosophy of the DHA program strategy. These new dental workers living and working in the medical clinics of those villages are an integrated part of the patient's medical/dental care team. As the DHA program continues to grow, educating and deploying more workers, they will become key elements of a medical/dental holistic approach to dental disease prevention.

ANTHC spearheaded the creation of the Alaska Dental Health Aide Program. They started by looking at the dental nurse/therapist education at Otago University in Dunedin, New Zealand, and at the dental therapist-training program in Canada. Otago University proved the most suitable choice for educating the first group of DHATs for Alaska. Three cohorts of students were sent to New Zealand for the 2-year educational program. However, changes in the New Zealand program, funding limitations, and the desire to provide dental therapy education closer to home led to the successful effort to establish a DHAT Educational Program based in the United States. Local training for the other levels of DHA has been available in Alaska since the inception of the DHA Initiative.

Over 50 countries utilize the dental therapist model as a strategy for improving oral health care access. The primary model for dental therapy programs worldwide is the dental therapist program in New Zealand. Starting in the 1920s, dental therapists (then called dental nurses) were educated and employed by the New Zealand government. The therapists worked in clinics in the schools, providing much improved access to the country's school children. The article “A Review of the Global Literature on Dental Therapist” provides a comprehensive overview of the impact of dental therapist globally. Due to the long history of the Dental Therapist in New Zealand, some of the most notable oral health changes have been documented in that country. For example, a 1970 New Zealand Medical Journal report showed that since 1923 the number of teeth needing extraction decreased from 88.2 per 100 children to 12.6 per 100 children. A 2003 report by the New Zealand National Health Committee stated that nearly all dental decay in New Zealand is either treated or restored by the end of the school year (6). In 2006, it was reported that ninety-seven percent of all children under the age 13 and 56% of all pre-schoolers in New Zealand were participating in the School Dental Service (7).

## Quality of care

The PDHA, EFDHA and DHAH all provide services that are allowed in other states, but prior to their introduction via the DHA Initiative, were not allowed in Alaska. The safety and quality record from appropriately educated and supervised providers similar to these in the United States is well documented. The DHATs were the first dental therapists in the United States, but had been used extensively in other countries since 1921. Virtually without exception, studies of dental therapists in other countries and expanded duty personnel in the United States show that these limited scope providers perform services they are educated to provide and certified or licensed to perform safely and competently. A study in Canada showed that the quality of restorations placed by dental therapists was equal and often better than those placed by dentists (8). A study completed in 2007, found through a chart audit that treatment provided by DHATs was within the scope of practice, delivered in a safe manner and met the standard of care of the dental profession (9). In a 2010 evaluation, Research Triangle International found that Alaska's dental health aide therapists provide safe and appropriate care (10).

## How dental health aides interact with dentists

Like the physician assistant model of medical care, which depends on a defined supervisory relationship between a physician and a physician assistant, a dentist supervises the dental health aides. Once matriculated, the dental health aides complete a preceptorship under direct supervision of experienced dentists before being certified and placed in remote villages. Once in the villages, dentists provide general supervision using telephone or telehealth technology. The dentists visit the villages periodically. The supervising dentist provides care beyond the scope of practice of the dental health aides in the village or the patient can be referred to more sophisticated regional centers or the Alaska Native Medical Center as part of this integrated system. This collaboration between providers with different scopes of practice is a very efficient way to provide care, and one which allows each provider to work up to the highest level of their education and certification or licensure. The dentist supervisor can spend his or her time on the more complicated patients for which they have more education to treat. The dental health aides provide the more basic services and pre-screen patients to insure the time the dentist spends with each patient can maximized. The patients benefit from increased access to higher level services which previously the dentists could not provide since they were providing less complicated services that DHAs can now provide. Quality assurance is critical and has been built into the system of DHA certification. The dentist, as the team leader, is the professional responsible for upholding the standards of practice through direct supervision of the DHA. Continuing education and recertification for all dental health aides is required. Recertification requires 24 hours of continuing education every 2 years and continual competency evaluation.

## Primary Dental Health Aide

The PDHA has 2 levels of certification. A PDHA I is able to provide fluoride varnish application, nutritional counselling, and oral hygiene instruction. A PDHA II can receive additional training in sealants, atraumatic restorative treatment, dental cleanings, dental radiology and/or dental assisting. Each course for PDHAs is 2 weeks in length.

## Expanded Function Dental Health Aide

The EFDHA can be broken down into 2 types and has 2 levels. The EFDHA I can be trained in basic restorative function and/or dental cleanings. The basic restorative function curriculum focuses on placing amalgam, composite and glass ionomer restorations in Class I, II, III and V cavity preparations. The dental prophylaxis course focuses on providing a supra-gingival cleaning. The EFDHA II is trained in advanced restorative function. This curriculum focuses on placing complex restorations.

## Dental Health Aide Hygienist

The DHAH was developed because hygienists working in Alaska were not allowed to administer local anaesthesia unless a dentist was physically present in the clinic with them. Delivery of care in the village is often completed by a dental hygienist after a dentist has completed treatment plans. The dental hygienist works off the dentist's treatment plan under general supervision after the dentist has left the village. If a patient needed scaling and root planning, the dental hygienist was not able to administer local anaesthesia because the dentist was no longer present in the clinic with them. Patients with more complex periodontal needs which required local anaesthesia were required to travel into the regional hub for treatment, even though a hygienist had traveled out to their village to provide care. Becoming certified as a DHAH allows a licensed dental hygienist with the appropriate training to provide local anaesthesia without a dentist being physically present in the clinic. Thus, patients are able to get their periodontal treatment needs met in their home community. The Alaska Dental Board Statues and Regulations were changed and hygienists are able, with appropriate training, to administer local anaesthesia without a dentist on site under collaborative care agreements.

## Dental Health Aide Therapist

Becoming certified as a DHAT requires the most education of all the DHAs. Students complete 2 years of post-high school education in dental disease prevention and basic dental treatment skills. Some of the courses covered include general health science, radiology, infection control, oral medicine, embryology, operative dentistry, local anaesthesia, cariology, pharmacology, diagnosis and treatment planning, and community prevention. Dental therapists are the only DHAs able to develop treatment plans for patient care. The DHAT has been the most controversial part of the DHA Program, due to the scope of practice including skills that were previously in the domain of only the dentist and because they are taught these skills in just 2 years without any other post-high school education required. A goal of the DHAT program is to get providers who will stay for long periods of time in underserved communities, thus improving continuity of care. It stands to reason that educating someone from the community to go back to the community would be more successful than recruiting people from the outside. The barrier has been that even with full scholarships available to AI/ANs to go to dental school, few have availed themselves of this opportunity. In Alaska’s rural communities, the graduation rates from high school are very low. Students may not view going to a 4-year college as a viable option due to various reasons including cost, geographic barriers and for some, the challenges associated with being a first-generation college student. The option of a 2-year certificate program is a more attainable goal for many of the recruits from rural Alaska communities. While dental therapy educational programs can be constructed in ways which include longer time to matriculate, the Alaska goals are better met by the proven 2-year post-high school model.

## Curriculum

The curriculum for the DHA education is required to meet rigorous standards set by the CHAP Certification Board. This board is a federally appointed board that oversees all Health Aide education and practice. The standards for education of health aides are outlined in the CHAP Certification Board Standards and Procedures document. For all DHA curricula, the standards clearly require the curriculum to meet the standards for similar providers in other parts of the country, and in the case of the DHAT, in other parts of the world. All approved curricula must be reviewed every 5 years by the CHAP Certification Board. In order to insure that the board is well informed in each type of practice, it has committees which assist in reviewing curricula and makes recommendations for the board. For the DHA curriculum, there is a Dental Academic Review Committee (DARC) which consists of experienced dentists, dental hygienists, a DHAT, and educators. Once a DARC review is completed, the DARC recommendations are presented to the CHAP Certification Board for consideration.

## Preceptorship, certification, and recertification

All DHAs are required to complete a preceptorship after successful completion of an approved educational program. During the preceptorship, the DHA works under the direct supervision (in the same clinic with the supervisor diagnosing and evaluating all work) of a dentist, or dental health aide therapist. Procedures must meet or exceed the minimum level of competency as determined by the supervisor. Each preceptorship has minimum requirements in terms of procedures and/or hours. A supervisor can choose to extend the length of the preceptorship as he or she sees necessary. The DHA applies for certification after completion of the educational program and preceptorship. The CHAP Certification Board reviews all applications. If they determine that the applications are complete and that requirements have been met, then the board can certify the DHA for 2 years.

Recertification of DHAs occurs every 2 years. In order to recertify, DHAs must complete 24 hours of continuing education and undergo a continual competency review by their supervisor.

## Prevention and basic care

The heart of the DHA Program is its strong foundation in prevention. The program clearly understands that in order to stop the epidemic of dental disease in AI/AN people living in Alaska, prevention must be aimed at changing behaviors in ways that are attainable and sustainable.

One key element running through the entire program is the belief that prevention must start early and be addressed in multiple ways. DHAs are partnering with Women, Infant and Children Programs, obstetric clinics, diabetes clinics, Head Start, schools, well-child clinics, and many other non-traditional sites for dental providers in order to increase the ability to provide prevention education and follow-up with patients after the initial encounter. Utilizing the motivational interviewing technique is the preferred method for working with patients on changing old habits to new, healthy habits. This technique engages the patients and allows them to drive their own individual prevention plan. The DHA becomes a partner in improving oral health outcomes with the patients, which is a critical departure from the traditional model used in dentistry for patient education.

By using the motivational interviewing technique, the provider engages the patient in a dialog about perceived obstacles to good oral health practices. Plans are formulated to work around these obstacles if possible, or perhaps the plan is modified to some smaller behavioral change that is attainable. Behavioral changes made in this way have been shown to be sustained for a longer period of time. Engaging the patient in this manner is respectful and builds trust. The result can be that the patient willingly returns for follow-up, which can lead to a better oral health outcome for the patient. This is also a way to ensure cultural competence because it requires respectful listening and engagement of the patient. DHAs are more likely to engage in motivational interviewing because this technique is emphasized throughout in their educational program.

## Conclusion

The DHA Program is an innovative local solution to a local problem. Since the inception of the DHA Initiative, over 45, 000 Alaskans can now access oral health care offered by a dental health aide. DHAs have been integrated into many of Alaska's Tribal Health Organizations dental and medical teams. DHAs allow for increased access to care and more efficient oral health care delivery. Culturally competent care is easier to achieve when DHA providers come from their home communities. The growth of the Dental Health Aide Program in Alaska is an integral part of helping Alaska Natives become the healthiest people in the world.
